# Combinational Therapy of Cardiac Atrial Appendage Stem Cells and Pyridoxamine: The Road to Cardiac Repair?

**DOI:** 10.3390/ijms22179266

**Published:** 2021-08-27

**Authors:** Lize Evens, Hanne Beliën, Sarah D’Haese, Sibren Haesen, Maxim Verboven, Jean-Luc Rummens, Annelies Bronckaers, Marc Hendrikx, Dorien Deluyker, Virginie Bito

**Affiliations:** 1 UHasselt—Hasselt University, BIOMED—Biomedical Research Institute, Agoralaan, 3590 Diepenbeek, Belgium; lize.evens@uhasselt.be (L.E.); hanne.belien@uhasselt.be (H.B.); sarah.dhaese@uhasselt.be (S.D.); sibren.haesen@uhasselt.be (S.H.); maxim.verboven90@gmail.com (M.V.); jeanluc.rummens@uhasselt.be (J.-L.R.); annelies.bronckaers@uhasselt.be (A.B.); marc.hendrikx@uhasselt.be (M.H.); dorien.deluyker@uhasselt.be (D.D.); 2 UHasselt—Hasselt University, Faculty of Medicine and Life Sciences, Agoralaan, 3590 Diepenbeek, Belgium

**Keywords:** stem cells, CASCs, advanced glycation end products, glycated proteins, myocardial infarction, transplantation, remodeling, cardiomyocytes, aldehyde dehydrogenase

## Abstract

Myocardial infarction (MI) occurs when the coronary blood supply is interrupted. As a consequence, cardiomyocytes are irreversibly damaged and lost. Unfortunately, current therapies for MI are unable to prevent progression towards heart failure. As the renewal rate of cardiomyocytes is minimal, the optimal treatment should achieve effective cardiac regeneration, possibly with stem cells transplantation. In that context, our research group identified the cardiac atrial appendage stem cells (CASCs) as a new cellular therapy. However, CASCs are transplanted into a hostile environment, with elevated levels of advanced glycation end products (AGEs), which may affect their regenerative potential. In this study, we hypothesize that pyridoxamine (PM), a vitamin B6 derivative, could further enhance the regenerative capacities of CASCs transplanted after MI by reducing AGEs’ formation. Methods and Results: MI was induced in rats by ligation of the left anterior descending artery. Animals were assigned to either no therapy (MI), CASCs transplantation (MI + CASCs), or CASCs transplantation supplemented with PM treatment (MI + CASCs + PM). Four weeks post-surgery, global cardiac function and infarct size were improved upon CASCs transplantation. Interstitial collagen deposition, evaluated on cryosections, was decreased in the MI animals transplanted with CASCs. Contractile properties of resident left ventricular cardiomyocytes were assessed by unloaded cell shortening. CASCs transplantation prevented cardiomyocyte shortening deterioration. Even if PM significantly reduced cardiac levels of AGEs, cardiac outcome was not further improved. Conclusion: Limiting AGEs’ formation with PM during an ischemic injury in vivo did not further enhance the improved cardiac phenotype obtained with CASCs transplantation. Whether AGEs play an important deleterious role in the setting of stem cell therapy after MI warrants further examination.

## 1. Introduction

Cardiovascular diseases are the leading cause of mortality worldwide, affecting 18 million people each year [1]. More than 40% of deaths related to cardiovascular diseases are due to myocardial infarction (MI). MI occurs when the coronary blood supply is interrupted, leading to irreversible loss of cardiomyocytes [2]. Following MI, adverse left ventricular (LV) remodeling often evolves into heart failure [3]. Current therapeutic approaches reduce the risk of recurrent infarctions and improve patient outcome. However, as lost cardiac tissue is not replaced [4], the progression towards heart failure with current therapies is only delayed, rather than prevented. As the renewal rate of the heart is limited [5], strategies to restore functional cardiac tissue are urgently needed. The ideal therapy should indeed replace necrotic cells while simultaneously restoring the function of viable tissue. In that context, a large number of endogenous stem cell types (such as mesenchymal stem cells (MSCs), endothelial progenitor cells (EPCs), and hematopoietic stem cells (HSCs)) have emerged as potential treatment options for regenerative medicine [6]. So far, cell-based approaches showed only minor improvements of cardiac function in pre-clinical models [7]. In patients, variable outcomes after stem cell therapy were observed. The latter are due to variations in patient response [8] or stem cell genetic modifications [9]. In addition, many randomized controlled trials failed to show improved functional and morphological outcome using these different stem cell types [10,11]. This discouraging result is undoubtedly related to the limited cardiomyogenic differentiation potential of the endogenous stem cell types used so far [10]. Resident cardiac stem cells (CSCs) are considered more suitable for myocardial regeneration. In contrast to other stem cells, they are most likely ‘pre-programmed’ to become cardiomyocytes. Several types of CSCs have been identified based on different markers (e.g., c-kit [12], Islet-1 [13]) or on the ability to form cardiospheres [14]. Nevertheless, the use of these specific CSCs showed only moderate improvements in clinical trials because of the limited capacity of CSCs to differentiate into new functional cardiomyocytes [15,16,17].

Our research group discovered a new type of cardiac stem cells, cardiac atrial appendage stem cells (CASCs) [18]. Identification of these stem cells is based on high aldehyde dehydrogenase (ALDH) enzyme activity. In vitro experiments have shown that the differentiation capacity of CASCs towards cardiomyocytes is superior to other CSCs types [18]. In addition, we have shown that autologous CASCs transplantation after MI improves global LV function [19]. This better cardiac outcome was associated with cell engraftment and CASCs’ differentiation in a cardiomyogenic phenotype [19]. Altogether, these data suggest a true high potential for using CASCs to repair lost cardiac tissue.

CASCs are transplanted after MI in a hostile environment of inflammation, fibrosis, and increased levels of advanced glycation end products (AGEs) [20]. AGEs are proteins and lipids that become glycated and oxidized after persistent contact with reducing sugars or short-chain aldehydes and/or a high degree of oxidative stress [21]. Next, to be abundantly present in our Western diet, accumulation of AGEs in the body is a natural process. This occurs with aging when the turnover rate of proteins is reduced. There is growing evidence reporting that AGEs contribute to the development and progression of cardiovascular dysfunction [22]. Indeed, increased circulating AGEs have been described to arise at an early lifetime in patients with cardiovascular diseases [23,24]. In ischemic heart disease patients, high levels of AGEs can also result from increased oxidative stress [22,25]. In addition, it is reported that immune cells (like neutrophils and macrophages) are mobilized to the ischemic area as a result of inflammation and cell death. These cells were shown to be major contributors to AGEs’ production [26,27]. These contribute to increased AGEs levels in patients suffering from MI. Recently, systematic review analysis [28] revealed that AGEs affect the viability and proliferation capacity of multiple types of stem cells in vitro, including CASCs [29], thereby affecting their therapeutic potential. These effects occur throughout several underlying mechanisms including excessive reactive oxygen species (ROS) generation, activation of the receptor for AGEs (RAGE), or via apoptotic pathways. As AGEs are increased in MI, we tested whether the regenerative capacities of CASCs could be further enhanced when combined with pyridoxamine (PM). PM is a compound able to reduce AGEs’ formation, a co-enzyme associated with multiple oxidative stress and inflammatory pathways and a strong iron chelator [30,31,32]. Using PM could thus potentially improve the efficiency of CASCs transplantation with no need to genetically modify them, in order to observe their full potential [33].

## 2. Results

### 2.1. AGEs’ Levels Are Reduced with PM Treatment

**Figure 1:**
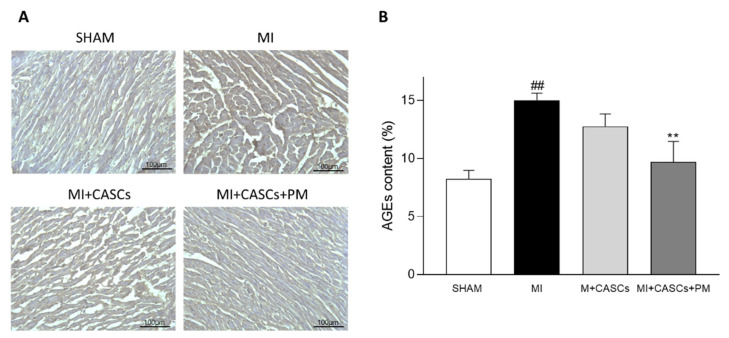
AGEs’ content in heart tissue is significantly decreased by PM. (**A**) Representative examples of transverse heart sections 4 weeks after surgery. The AGEs’ content (brown) was immunohistologically determined with DAB staining. Scale bar = 100 µm. (**B**) Quantification of AGEs’ content in hearts from SHAM (*n* = 4), MI (*n* = 11), MI + CASCs (*n* = 10), and MI + CASCs + PM (*n* = 5). Data are expressed as mean ± SEM. ** denotes *p* < 0.01 vs. MI and ## denotes *p* < 0.01 vs. SHAM.

Total AGEs’ levels were measured in heart tissues from SHAM, MI, MI + CASCs, and MI + CASCs + PM, and representative images are provided in Figure 1A. AGEs’ content was significantly increased in MI animals compared with SHAM (Figure 1B; 15% ± 0.6 in MI vs. 8.2% ± 0.7 in SHAM) and PM significantly decreased the AGEs’ content compared with MI (Figure 1B; 9.7% ± 1.8 in MI + CASCs + PM).

### 2.2. CASCs Transplantation Prevents Loss of LV Function after MI

In vivo cardiac function was assessed by echocardiographic and hemodynamic measurements. Representative examples of echocardiographic images of SHAM, MI, MI + CASCs, and MI + CASCs + PM 4 weeks post-operative are shown in Figure S1. Echocardiographic parameters of the different groups, SHAM, MI, MI + CASCs, and MI + CASCs + PM, 4 weeks post-operative are summarized in Table 1. Additional echocardiographic parameters are summarized in Table S1. MI animals undergoing CASCs transplantation with or without additional PM treatment displayed a significantly increased ejection fraction (EF) compared with MI (Table 1; 59% ± 4 in MI vs. 79% ± 3 in MI + CASCs; vs. 72% ± 3 in MI + CASCs + PM, *p* = 0.051).

Hemodynamic measurements of SHAM, MI, MI + CASCs, and MI + CASCs + PM were performed 4 weeks after surgery. Compared with MI, additional PM treatment significantly reduced the time constant for isovolumetric relaxation (Table 2; Tau; 0.0499 s ± 0.017 in MI vs. 0.0117 s ± 0.001 in MI + CASCs + PM).

### 2.3. CASCs Transplantation Tended to Reduce Infarct Size

**Figure 2:**
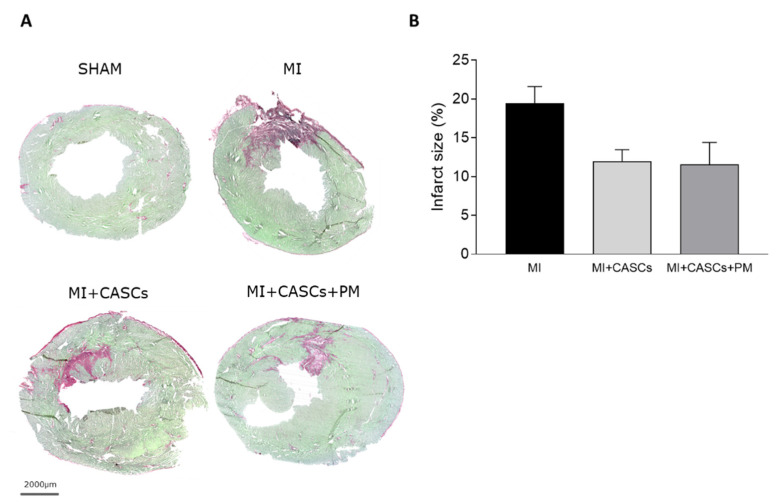
Assessment of infarct size. (**A**) Representative examples of hearts from SHAM, MI, MI + CASCs, and MI + CASCs + PM. Fibrotic tissue, as a surrogate for infarct size, is stained red, while viable tissue is stained green. Scale bar = 2000 µm. (**B**) Quantification of infarct size in transversal sections 4 weeks post-surgery. MI (*n* = 11), MI + CASCs (*n* = 10), and MI + CASCs + PM (*n* = 5). Data are expressed as mean ± SEM.

Figure 2A demonstrates representative examples of Sirius Red/Fast Green stained cryosections from SHAM, MI, MI + CASCs, and MI + CASCs + PM 4 weeks after surgery. Infarct size tended to decrease in animals undergoing CASCs transplantation (Figure 2B; 19% ± 2 in MI vs. 12% ± 2 in MI + CASCs, *p* = 0.07). Additional PM treatment did not further reduce infarct size (Figure 2B; 12% ± 3 in MI + CASCs + PM, *p* = 0.16).

### 2.4. CASCs Transplantation Prevents the Increased Interstitial Collagen Deposition Seen with MI

**Figure 3:**
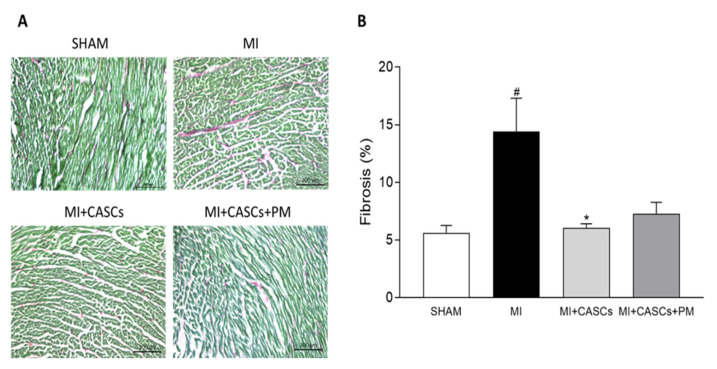
Interstitial collagen deposition in the LV. (**A**) Representative examples of collagen deposition (red) in the LV. Scale bar = 200 µm. (**B**) Quantification of collagen content in LV transversal sections 4 weeks after surgery of SHAM (*n* = 4), MI (*n* = 11), MI + CASCs (*n* = 10), and MI + CASCs + PM (*n* = 5). Data are expressed as mean ± SEM. * denotes *p* < 0.05 vs. MI, # denotes *p* < 0.05 vs. SHAM.

Representative images of interstitial collagen obtained with Sirius Red/Fast Green staining in LV sections from the groups are provided in Figure 3A. Interstitial collagen deposition was significantly lower in CASCs transplanted animals compared with MI (Figure 3B; 14% ± 3 in MI vs. 6% ± 0.4 in MI + CASCs). Fibrosis tended to be lower in PM animals (Figure 3B; 7% ± 1 in MI + CASCs + PM).

### 2.5. CASCs Transplantation Prevents Resident Cardiomyocyte Functional Remodeling

**Figure 4:**
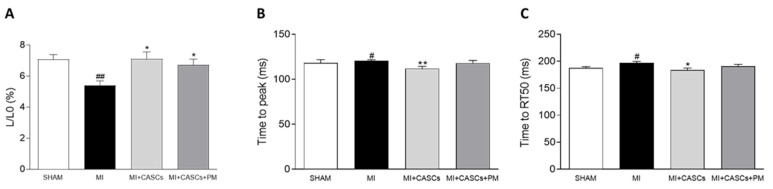
Resident cardiomyocyte shortening during field stimulation at 4 Hz. Quantification of (**A**) unloaded cell shortening normalized to diastolic cell length (L/L_0_), (**B**) time to peak of contraction (TTP), and (**C**) time to half-maximal relaxation (RT_50_) of resident cardiomyocytes from SHAM (*n*_cells_ = 80; *n*_animals_ = 7), MI (*n*_cells_ = 60; *n*_animals_ = 5), MI + CASCs (*n*_cells_ = 41; *n*_animals_ = 4), and MI + CASCs + PM (*n*_cells_ = 31; *n*_animals_ = 3). Data are expressed as mean ± SEM. * denotes *p* < 0.05; ** denotes *p* < 0.01 vs. MI, # denotes *p* < 0.05 vs. SHAM, and ## denotes *p* < 0.01 vs. SHAM.

Unloaded cell shortening was measured in freshly isolated cardiomyocytes isolated from SHAM, MI, MI + CASCs, and MI + CASCs + PM animals 4 weeks post-surgery. As shown in Figure 4A, cells isolated from the border zone of infarcts displayed altered functional properties, namely reduced and slower unloaded cell shortening. CASCs transplantation, with or without PM treatment, could prevent the deterioration of the cardiomyocyte contractile properties (Figure 4A; L/L_0_, 5% ± 0.3 in MI vs. 7% ± 0.5 in MI + CASCs; vs. 7% ± 0.4 in MI + CASCs + PM). The kinetics of cell contraction and cell relaxation, i.e., TTP and RT_50_, were significantly better in MI + CASCs and tended to be improved in MI + CASCs + PM animals (Figure 4B TTP; 120 ms ± 1 in MI vs. 112 ms ± 2 in MI + CASCs; vs. 118 ms ± 3 in MI + CASCs + PM; Figure 4C RT_50_; 197 ms ± 3 in MI vs. 184 ms ± 4 in MI + CASCs; vs. 190 ms ± 4 in MI + CASCs + PM).

### 2.6. PM Treatment Tended to Reduced Tissue Pro-Inflammatory Cytokine Levels

**Figure 5:**
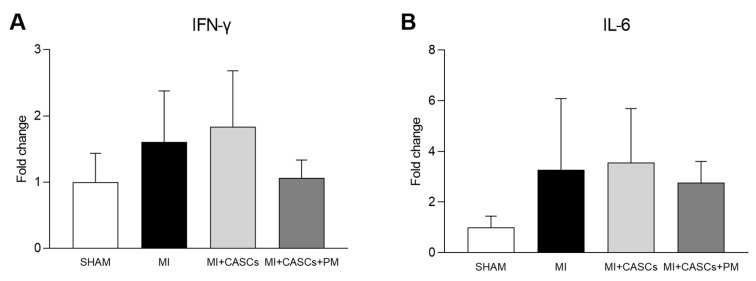
Gene expression of inflammatory cytokines. Quantification of gene expression of (**A**) IFN-γ and (**B**) IL-6 in LV tissue from SHAM (*n* = 4), MI (*n* = 7), MI + CASCs (*n* = 7), and MI + CASCs + PM (*n* = 3). Data are expressed as mean ± SEM.

Gene expressions of pro-inflammatory cytokines (IFN-γ and IL-6) were evaluated in the four groups of animals, 4 weeks post-surgery. As shown in Figure 5, the expression of inflammatory cytokines tended to be lower in MI + CASCs + PM animals compared with MI and MI + CASCs animals (Figure 5A IFN-γ; 1.60 ± 0.27 in MI vs. 1.83 ± 0.30 in MI + CASCs; vs. 1.06 ± 0.14 in MI + CASCs + PM; Figure 5B IL-6; 3.28 ± 1.06 in MI vs. 3.56 ± 0.81 in MI + CASCs; vs. 2.77 ± 0.48 in MI + CASCs + PM).

## 3. Discussion

In our study, we have shown that, after MI, CASCs transplantation is able to improve the cardiac phenotype by limiting cellular remodeling. However, preventing AGEs’ formation did not further enhance the positive outcome provided by CASCs alone.

### 3.1. Combining CASCs and PM to Enhance Cardiac Repair

Oxidative stress is one of the main factors inducing AGEs’ synthesis, by formation of reactive carbonyl compounds and glycoxidation of Amadori products in the Maillard reaction. In patients with MI, AGEs’ levels are significantly increased [25,34] and have potential deleterious effects on cardiac function [35,36]. Moreover, in our study, AGEs are significantly increased in MI animals. In addition to increased oxidative stress, the inflammatory process induced after MI activates neutrophils and macrophages. These immune cells are known to further secrete AGEs and are reported to be key inducers of AGEs’ formation in MI [26,27].

Even if they provide new insights into tissue regeneration, stem cells are transplanted in the border zone of MI with increased oxidative stress, inflammation, and AGEs’ levels. Previous studies have shown that increased levels of AGEs affect stem cells’ properties, i.e., by reducing their proliferation and migration properties [28]. Recently, we have demonstrated that the same adverse effects of AGEs apply to CASCs’ properties in vitro [29]. In that context, reducing AGEs’ formation could potentially enhance CASCs’ regenerative properties upon in vivo transplantation. To find out whether such an approach would offer new therapeutic insights, was the goal of our study. In the context of improving stem cell therapy by targeting AGEs, we evaluated the effect of CASCs transplantation in combination with PM in a rat model of MI. This vitamin B derivate is an effective and safe AGEs-lowering therapy [37], which has different mechanisms of action [38,39]. First, PM can bind with catalytic redox metal ions, which are needed for glycoxidation of Amadori products. As such and related to its iron-chelator properties, Amadori-to-AGEs conversion is blocked. Secondly, PM can scavenge reactive carbonyl compounds, the latter being major AGEs’ precursors. In addition, studies have demonstrated that PM is a co-enzyme associated with multiple inflammatory pathways, thereby potentially inhibiting inflammation [32]. Finally, by inhibiting ROS formation or scavenging oxygen radicals, PM has been shown to be a potent antioxidant. Previous studies have shown that, even independent of stem cell transplantation, PM alone improves survival and reduces extracellular remodeling after MI, by reducing AGEs’ levels [40]. Moreover, in clinical trials, PM has been demonstrated as a safe and effective drug in diabetic patients [38]. However, owing to financial issues, a clinical trial of NephroGenex in 2014, testing PM as an anti-diabetic treatment, was stopped [41]. No other clinical trials are currently investigating PM as a therapy.

In our study, PM succeeded in reducing total AGEs’ tissue levels. However, PM did not further improve cardiac function obtained with CASCs transplantation alone, which was, at first sight, not expected in our study. This could be partially explained by the recent discovery of Vagnozzi et al. [42]. In their article, the authors show that cellular therapy itself induces an inflammatory response after MI that could be the primary beneficial effect underlying stem cell treatment. Pro-inflammatory macrophages, mobilized and activated by transplanted stem cells, could indeed rejuvenate the mechanical properties of the injured cardiac area. By affecting fibroblast activity, the ECM content and area occupied by scar tissue could be reduced. The precise underlying mechanisms responsible for the repair response of these immune cells are unclear and require further investigation. In our study, it is thus likely that, by reducing local inflammation, PM could not further enhance the repair process, when combined with CASCs transplantation. Inflammatory cytokines such as IFN-γ and IL-6 tended to be reduced by PM treatment in our study. As an immune reaction is thus needed as a base for would healing with stem cells, PM could counteract the positive effects of CASCs therapy by reducing local tissue inflammation. This could explain the lack of additive value of PM in the context of MI and stem cell transplantation.

Even if our data do not demonstrate an additional effect of PM to CASCs transplantation, the potential of other anti-AGEs therapies still needs to be investigated. It has been shown in Alzheimer’s, Parkinson’s, and rheumatoid arthritis disease animal models that stem cell survival was prolonged, migration capacity was enhanced, and the MSCs were better protected against apoptosis, when sRAGE-secreting MSCs were transplanted. By scavenging AGEs with sRAGE, the effectiveness of MSCs transplantation was improved, thus suggesting a role of AGEs in regenerative approaches with stem cells [43,44,45]. However, using genetically modified stem cells is still highly experimental and needs to be investigated in vivo before any possible translation into the clinical setting is possible. Therefore, one cannot exclude that other anti-AGEs therapy approaches such as RAGE inhibitors, sRAGE, or ALT-711 could potentially succeed in further lowering AGEs’ concentrations in MI and potentially have an additive effect on cardiac outcome. These therapeutic options need to be investigated in both pre-clinical and clinical studies in combination with CASCs therapy.

### 3.2. CASCs Alone Are an Effective Therapy for MI

Independent of PM treatment, our data confirm that transplantation of CASCs can prevent worsening of cardiac function after an ischemic injury, as shown by Fanton et al. in the minipig model [19]. Indeed, we have shown that EF significantly increased up to 20% after CASCs transplantation compared with non-treated animals. Meta-analysis of other CSCs therapies for the treatment of MI in mice showed an overall increase in EF of 9.9% [46]. Therefore, CASCs do have more effective regenerative effects compared with other CSCs and are remarkable candidates for cellular therapy. Other parameters of global cardiac function, such as dP/dt_max_, SV, CO, and ESV, even if not significantly affected, followed the same trend, indicating an overall improvement of systolic function upon CASCs transplantation. In addition, infarct size tended to decrease after CASCs transplantation. Furthermore, as shown by the prevention of adverse remodeling at the cardiomyocyte level, our data suggest that mechanical load subjected to the resident myocytes of the ischemic area was reduced with CASCs transplantation. The prevention of collagen deposition seen in our study is also in line with a potentially reduced mechanical load with CASCs transplantation. Indeed, an important pathway in post-MI remodeling and scar formation is the TGF-β1 signaling pathway. In this study, we did not evaluate the underlying mechanisms resulting in reduced fibrosis with CASCs. However, TGF-β1 could be an essential contributor. Indeed, increased TGF-β1 is detected after MI and is known to decrease the expression and function of enzymes responsible for matrix degradation and increase the inhibitors of proteases [47,48]. Whether CASCs transplantation results in a reduced lysyl oxidase expression and/or PI3K/Akt, Smad3, and MAPK signaling pathway as a consequence of increased TGF-β1 activation [49], remains to be confirmed. However, other studies have shown that MSCs transplantation is able to ameliorate cardiac fibrosis by decreasing TGF-β1 levels [50]. Therefore, it seems likely that this TGF-β1 pathway is also involved in our study as it is a common pathway found in many diseases, but this has to be confirmed. In addition, we have demonstrated that CASCs transplantation can prevent adverse cellular remodeling of resident cardiomyocytes, isolated from the border zone of the infarct. Indeed, we show that, compared with MI animals, the amplitude and kinetics of cardiomyocyte shortening isolated from the border zone of MI in transplanted animals are improved. In that context, it has been described that the extent of mechanical load determines the extent of remodeling in both peri-infarct and remote regions [51]. It is then very likely that even a small decrease in infarct size, which we observe upon CASCs transplantation, will reduce adverse cellular remodeling in the resident cardiomyocytes.

However, whether the beneficial cardiac outcome is solely attributed to new cardiomyocytes differentiated from CASCs or to the paracrine factors secreted by these stem cells remains to be investigated. This then raises the question of whether CASCs were still present 4 weeks after transplantation. Indeed, pre-clinical studies have shown that most of the stem cells injected at the site of injury are cleared out within seconds, resulting in only 1–3% of the injected cells persisting at the site of injury [52]. Yet, previously, the presence of differentiated CASCs 8 weeks post-MI was demonstrated by immunostainings [19]. In addition, engraftment of the CASCs after 8 weeks of acute MI has been shown to be 19%, a value higher than that previously described with other stem cells [19]. It is thus very likely that CASCs are still present in the cardiac tissue 4 weeks post-transplantation. In addition, previous studies have shown that stem cells are able to secrete paracrine factors to promote survival and proliferation or have immunomodulatory effects on resident cardiomyocytes [53]. The strong paracrine effects of stem cells have been well documented and are, for a part, related to the limited improvement of cardiac function seen in some studies, as those may compensate for the lack of cardiomyocyte differentiation [54,55]. Whether the differentiation of CASCs to new cardiomyocytes and/or the paracrine factors secreted by the CASCs are the reason for the improvements seen after MI, remains to be clarified.

## 4. Materials and Methods

### 4.1. Animal Experiments

Animal studies were conducted in accordance with the EU Directive 2010/63/EU for animal experiments and were approved by the Local Ethical Committee for Animal Experimentation (UHasselt, Belgium, Diepenbeek; ID 201701K & ID 202050). All animals were kept in a temperature-controlled environment (21 °C, 60% humidity) with a 12 h/12 h light/dark cycle. They were fed a standard pellet diet with water available *ad libitum*. In total, 72 female Sprague-Dawley rats (Janvier Labs, Le Genest-Saint-Isle, France) were used for the in vivo animal experiments. Twenty-nine female Sprague-Dawley rats (Janvier Labs) were used for the CASCs isolation, expansion, and transplantation.

### 4.2. Rat CASCs Isolation and Expansion

CASCs were harvested from the right atrial appendages, as described before [18]. Briefly, rats were injected with heparin (1000 u/kg, intraperitoneally (*i.p.*)) and were euthanized with an overdose of sodium pentobarbital (Dolethal, Vetoquinol, Aartselaar, Belgium, 200 mg/kg, *i.p.*). Hearts were harvested and perfused with a normal Tyrode solution (137 mM NaCl, 5.4 mM KCl, 0.5 mM MgCl_2_, 1 mM CaCl_2_, 11.8 mM Na-HEPES, 10 mM glucose, 20 mM taurine, pH 7.4), and the right atrial appendages were collected. The extracted right atrial appendage tissue was minced into pieces of ~1 mm^3^, washed with phosphate buffered saline (PBS), and enzymatically dissociated for 30 min in Hank’s balanced salt solution containing 0.6 WU/mL collagenase NB 4 (Serva, Heidelberg, Germany) and 20 mM CaCl_2_. Next, ALDH^+^ cells were stained according to the Aldefluor kit (STEMCELL Technologies, Evergem, Belgium). ALDH^+^ cells were defined as CASCs and were flow-sorted (BD FACS Aria) in X-VIVO 15 media (Lonza, Basel, Switzerland) supplemented with 20% fetal calf serum (FCS) and 2% penicillin/streptomycin (P/S). Isolated CASCs were seeded in six-well plates at a density of 60,000 cells per well and incubated at 37 °C in a humidified incubator with a 5% CO_2_ atmosphere. Medium was changed every 2 to 3 days. When CASCs reached 80% confluence, they were harvested using trypsin. For all experiments, passage 1 CASCs were used.

### 4.3. Experimental Protocol

Rats were randomly assigned into four groups undergoing surgery: SHAM, MI, MI with CASCs transplantation (MI + CASCs), and MI with CASCs transplantation undergoing additional PM treatment (MI + CASCs + PM). PM treatment (1 g/L in drinking water) was started 1 week prior to surgery [40]. MI rats were subjected to left anterior descending coronary artery (LAD) ligation, as described previously [40]. The mortality rate after LAD occlusion in all MI groups was 35%, resulting in 39 surviving animals. In brief, rats were anesthetized using 2% isoflurane supplemented with oxygen, intubated via a transversal incision in the trachea and mechanically ventilated. A left thoracotomy was performed in the intercostal space between the third and fourth ribs to expose the heart. The pericardium was opened, and the thymus was partially removed. The LAD was occluded with 6/0 Prolene suture (Ethicon, Deforce Medical, Ardooie, Belgium). Successful occlusion was confirmed by LV pallor immediately after ligation. Rats undergoing MI received either no injections (MI, *n* = 16) or intramyocardial injections, containing either 2 × 10^6^ CASCs (MI + CASCs, *n* = 14) or 2 × 10^6^ CASCs with PM (MI + CASCs + PM, *n* = 9). CASCs were harvested at passage 1 for transplantation. Briefly, CASCs were centrifuged for 5 min at 1200 rpm and resuspended at a density of 2 × 10^6^ cells in a Matrigel construct containing 44.4% X-VIVO media supplemented with 10% FCS, 2% P/S, 2% Amphotericin B, 34% collagen type I, 16% Matrigel, and 1.7% NaHCO_3_. CASCs were kept on ice in a 29-gauge needle until being used for transplantation. Intramyocardial injections were performed with a maximal total volume of 150 µL at three different points around the peri-infarct zone. After transplantation, the chest was closed, and the lungs were re-inflated. After restoration of spontaneous respiration, the animal was extubated and the trachea was closed. SHAM animals (*n* = 12) underwent the same surgical procedure without LAD ligation and without CASCs transplantation. The non-steroidal anti-inflammatory drug meloxicam (Metacam, Boehringer Ingelheim Vetmedica GmbH, Rohrdorf, Germany, 1 mg/kg, subcutaneously) was administered post-operatively once a day for 2 consecutive days. Non-invasive echocardiographic and invasive hemodynamic measurements were performed at sacrifice. After harvesting the hearts, single cardiomyocytes were isolated from the injection zone in the LV. In addition, transversal sections of the LV were fixed in 4% PFA for 24 h and placed in 30% sucrose for cryopreservation. After 24 h, transversal sections were embedded in frozen section compound (Leica Microsystems, Amsterdam, The Netherlands) for storage at −80 °C until staining. Residual tissue of the LV was crushed to a fine powder, immediately snap-frozen in liquid nitrogen, and stored at −80 °C for further real-time PCR analysis.

### 4.4. Echocardiographic Measurements

Transthoracic echocardiographic images were obtained from all animals under 2% isoflurane anesthesia supplemented with oxygen 4 weeks post-operative with a Vevo 3100 system and a 21 MHz linear probe MX250 (FUJIFILM VisualSonics Inc., Amsterdam, The Netherlands), as described previously [56]. Rats were placed in a supine position, the thorax was shaved, and depilatory cream was applied to prevent hair-based artifacts. Heart rate, respiratory rate, and ECG signals were monitored while measurements were taken, using the accompanying Vevo Imaging Station. Parasternal long-axis and short-axis views at mid-ventricular level were acquired in both B-mode and M-mode. Four-dimensional images were acquired. The apical four-chamber view was used to obtain mitral inflow profiles by pulsed wave Doppler for estimation of diastolic function. In addition, diastolic annular velocities were captured by tissue Doppler imaging at the septal mitral annulus. Echocardiographic images were analyzed using the Vevo Lab 3.2.6 software (FUJIFILM VisualSonics Inc.). Standard measures of systolic function, diastolic function, and LV structure were analyzed. To reduce bias, analysis of the echocardiographic data was blinded.

### 4.5. Hemodynamic Measurements

Invasive hemodynamic measurements were obtained at sacrifice under 2% isoflurane anesthesia supplemented with oxygen, as described before [57]. Hemodynamic parameters were measured with an SPR-320 Mikro-Tip single pressure catheter (Millar Inc., The Hague, The Netherlands) placed into the LV via the right carotid artery. The catheter was connected to a quad-bridge amplifier and PowerLab26T module (AD Instruments, Oxford, United Kingdom) to transfer the pressure data to LabChart v7.3.7 software (AD Instruments). Hemodynamic parameters were obtained from this software (peak rate of pressure rise (dP/dt_max_), peak rate of pressure decline (dP/dt_min_), and time constant for isovolumetric relaxation (Tau)).

### 4.6. Cardiomyocyte Isolation and Unloaded Cell Shortening

Four weeks after surgery, rats were injected with heparin (1000 u/kg, *i.p.*) and euthanized by injection with an overdose of sodium pentobarbital (Dolethal, Vetoquinol, 200 mg/kg, *i.p.*). Hearts were harvested and single cardiomyocytes were isolated from the LV by enzymatic dissociation through retrograde perfusion of the aorta, as described previously [58,59]. The hearts were perfused for 1 min with a normal Tyrode solution and then connected to a Langendorff perfusion system for following perfusion steps at 37 °C and 100% O_2_ oxygenation. Perfusion with a Ca^2+^-free solution (130 mM NaCl, 5.4 mM KCl, 1.2 mM KH_2_PO_4_, 1.2 mM MgSO_4_, 6 mM HEPES, 20 mM glucose, pH 7.2) was performed for 8 min, followed by perfusion with an enzyme solution (Ca^2+^-free solution containing 1.5 g/L collagenase type II (Worthington, Brussels, Belgium) and 0.06 g/L protease type XIV (Sigma-Aldrich, Overijse, Belgium)) for variable time periods (12–20 min). Finally, hearts were perfused with a low Ca^2+^ solution (Ca^2+^-free solution containing 0.1 mM CaCl_2_ and 20 mM taurine) for 5 min. The digested LV tissue was minced and filtered, after which Ca^2+^ concentration was gradually increased to 1 mM with normal Tyrode solution. Unloaded cell shortening experiments were performed on intact cardiomyocytes in normal Tyrode at room temperature. Measurements were performed on cardiomyocytes from the remote border zone of the infarct of MI-operated animals. The cardiomyocytes from SHAM animals were also isolated from the same area in the LV. Cardiomyocyte shortening was measured with a video-edge detector (Crescent Electronics, London, UK) during field stimulation with constant pulses above the threshold at 1, 2, and 4 Hz using platinum electrodes. Unloaded cell shortening was normalized to diastolic cell length (L/L_0_, %). The kinetics of cell shortening were assessed by measuring time to peak of contraction (TTP, ms) and time to half-maximal relaxation (RT_50_, ms).

### 4.7. AGEs’ Content in Heart Tissue

Transversal frozen sections of 10 µm were obtained and immunohistologically stained for AGEs with DAB staining. First, antigen retrieval was performed with citrate buffer (pH = 6). The sections were blocked for 1 h at room temperature with serum-free protein block (X0909, Dako Agilent, Diegem, Belgium). Tissue sections were incubated overnight at 4 °C with a rabbit anti-rat primary antibody for AGEs (1/250, Abcam, ab23722). EnVision™ + Dual Link System-HRP (Dako Agilent, anti-rabbit/anti-mouse, K4061) was applied for 30 min at room temperature. DAB solution was added (Dako Agilent) and sections were counterstained with hematoxylin. Coupes were dehydrated and mounted with DPX mounting medium. Negative controls were included in each staining, in which the staining procedure was performed with omission of the primary antibody. Images were acquired using a Leica MC170 camera connected to a Leica DM2000 LED microscope. The AGEs deposition was quantified with Fiji/ImageJ software (1.53c) in four randomly chosen regions. The AGEs-positive area was normalized to the total cardiomyocyte area and expressed as AGEs’ content in %.

### 4.8. Interstitial Fibrosis and Infarct Size Measurement

Transversal frozen tissue sections of 10 µm were obtained and stained according to the Sirius Red/Fast Green Collagen Staining Kit for frozen sections (Chondrex Inc., Redmond, WA, USA). After staining, sections were dehydrated in increasing concentrations of ethanol, followed by a xylene wash, and mounted in DPX mounting medium. Collagenous tissue stains red, while non-collagenous tissue stains green. Interstitial fibrosis was measured in four randomly chosen 10× zoomed-in images, obtained in the peri-infarct zone and LV remote region using a Leica DM2000 LED microscope (Leica Microsystems). Infarct size was assessed on images of whole tissue slides using the AxioScan (Zeiss, Zaventem, Belgium). The percentage of interstitial collagen deposition and infarct size was assessed using the color deconvolution plugin in Fiji/ImageJ software (1.53c) [60] and was expressed as % of the total surface area of interest.

### 4.9. Real-Time PCR

As described previously [61], total RNA was extracted from ±30 mg snap-frozen LV tissue using RNeasy fibrous tissue kit (Qiagen, Venlo, The Netherlands) following the manufacturer’s guidelines. The concentration and purity of RNA were assessed with the NanoDrop 2000 spectrophotometer (Isogen Life Science, Utrecht, The Netherlands). RNA was reverse-transcribed to cDNA using the qScript cDNA SuperMix (Quantabio, VWR, Leuven, Belgium). The expressions of interferon-γ (IFN-γ) and interleukin-6 (IL-6) were studied. Primers (Table S2) were designed in the coding sequence of the mRNA. Real-time PCR was carried out in a MicroAmp Fast Optical 96-well reaction plate (Thermo Fisher Scientific, Merelbeke, Belgium) using the QuantStudio 3 Real-Time PCR System (Thermo Fisher Scientific). SYBR Green (Thermo Fisher Scientific) chemistry-based qPCR was performed [62]. Gene expression data were analyzed via the ΔΔCt method with consideration of the MIQE guidelines [63]. The most stable reference genes for this experimental set-up were determined by geNorm (hypoxanthine-guanine phosphoribosyl transferase (HPRT) and phosphoglycerate kinase 1 (PGK1); Table S2).

### 4.10. Statistics

Statistical analyses were performed using GraphPad Prism 9.0.0 software (San Diego, CA, USA). Normal distribution of data was assessed with the Shapiro–Wilk test. Experimental data that were normally distributed were subjected to a one-way ANOVA followed by Bonferroni’s multiple comparisons test. If data were not normally distributed, Kruskal Wallis followed by Dunn’s multiple comparisons test was used. All data are expressed as mean ± standard error of the mean (SEM). A value of *p* < 0.05 was considered statistically significant.

## 5. Conclusions

We have demonstrated in a ratmodel of MI that transplantation of CASCs can prevent worsening of cardiac function after an ischemic injury. These findings are an important stepping stone towards the use of CASCs as an effective stem cell therapy in the clinic. However, additional PM treatment and reduced tissue AGEs’ levels did not display added value when combined with CASCs transplantation. Whether or not other anti-AGEs therapies specifically targeting inflammation combined with CASCs therapy could have beneficial effects cannot be excluded, and remains to be further investigated.

## Figures and Tables

**Table 1:** Echocardiographic characteristics.

Parameters	4 Weeks Post-Operative			
	SHAM	MI	MI + CASCs	MI + CASCs + PM
EF (%)	80 ± 4	59 ± 4 ^##^	79 ± 3 ***	72 ± 3
HR (bpm)	333 ± 14	318 ± 12	332 ± 12	327 ± 13
SV (µL)	170 ± 16	162 ± 17	172 ± 17	192 ± 17
CO (mL/min)	57 ± 4	51 ± 5	59 ± 6	65 ± 6
EDV (µL)	215 ± 26	298 ± 51	217 ± 17	267 ± 22
ESV (µL)	45 ± 13	136 ± 36	44 ± 5 *	77 ± 11
AWT (mm)	1.74 ± 0.13	1.55 ± 0.16	1.65 ± 0.12	1.80 ± 0.16
PWT (mm)	1.54 ± 0.16	1.63 ± 0.15	1.57 ± 0.09	1.62 ± 0.17

Echocardiographic characteristics 4 weeks post-surgery in SHAM (*n* = 5), MI (*n* = 11), MI + CASCs (*n* = 10), and MI + CASCs + PM (*n* = 9) animals. Data are expressed as mean ± SEM. * denotes *p* < 0.05, *** denotes *p* < 0.001 vs. MI, and ^##^ denotes *p* < 0.01 vs. SHAM. EF: ejection fraction, HR: heart rate, SV: stroke volume, CO: cardiac output, EDV: end-diastolic volume, ESV: end-systolic volume, AWT: anterior wall thickness, PWT: posterior wall thickness.

**Table 2:** Hemodynamic characteristics.

Parameters	4 Weeks Post-Operative			
	SHAM	MI	MI + CASCs	MI + CASCs + PM
Max LV pressure (mmHg)	99 ± 3	90 ± 2	96 ± 3	103 ± 4 *
dP/dt_max_ (mmHg/s)	6773 ± 529	6038 ± 242	6923 ± 340	6551 ± 257
dP/dt_min_ (mmHg/s)	−7269 ± 683	−6550 ± 705	−6816 ± 354	−6917 ± 273
Tau (s)	0.0130 ± 0.001	0.0499 ± 0.017	0.0148 ± 0.002	0.0117 ± 0.001 **

Hemodynamic characteristics 4 weeks post-op of SHAM (*n* = 5), MI (*n* = 9), MI + CASCs (*n* = 10), and MI + CASCs + PM (*n* = 9) animals. Data are expressed as mean ± SEM. * denotes *p* < 0.05 and ** denotes *p* < 0.01 vs. MI. LV: left ventricular, dP/dt_max_: peak rate of pressure rise, dP/dt_min_: peak rate of pressure decline, Tau: time constant for isovolumetric relaxation.

## Data Availability

The data presented in this study are available on request from the corresponding author.

## Supplementary Materials

The following are available online at https://www.mdpi.com/article/10.3390/ijms22179266/s1.
