# Supplementary material for: Combinational Therapy of Cardiac Atrial Appendage Stem Cells and Pyridoxamine: The Road to Cardiac Repair?
Source: Int J Mol Sci. 2021 Aug 27;22(17):9266. doi: 10.3390/ijms22179266 (PMC8431115; doi:10.3390/ijms22179266)
Supplement: Supplementary file 1 [file ijms-22-09266-s001.zip › ijms-1298933-supplementary.pdf]

Supplementary Material

Combinational Therapy of Cardiac Atrial Appendage Stem Cells and Pyridoxamine: The Road to Cardiac Repair?

Lize Evens [1], Hanne Beliën [1], Sarah D'Haese [1], Sibren Haesen [1], Maxim Verboven [1], Jean-Luc Rummens [1,2], Annelies Bronckaers [1], Marc Hendrikx [1], Dorien Deluyker [1,†], and Virginie Bito [1,*,†]

1. Supplemental figures & tables

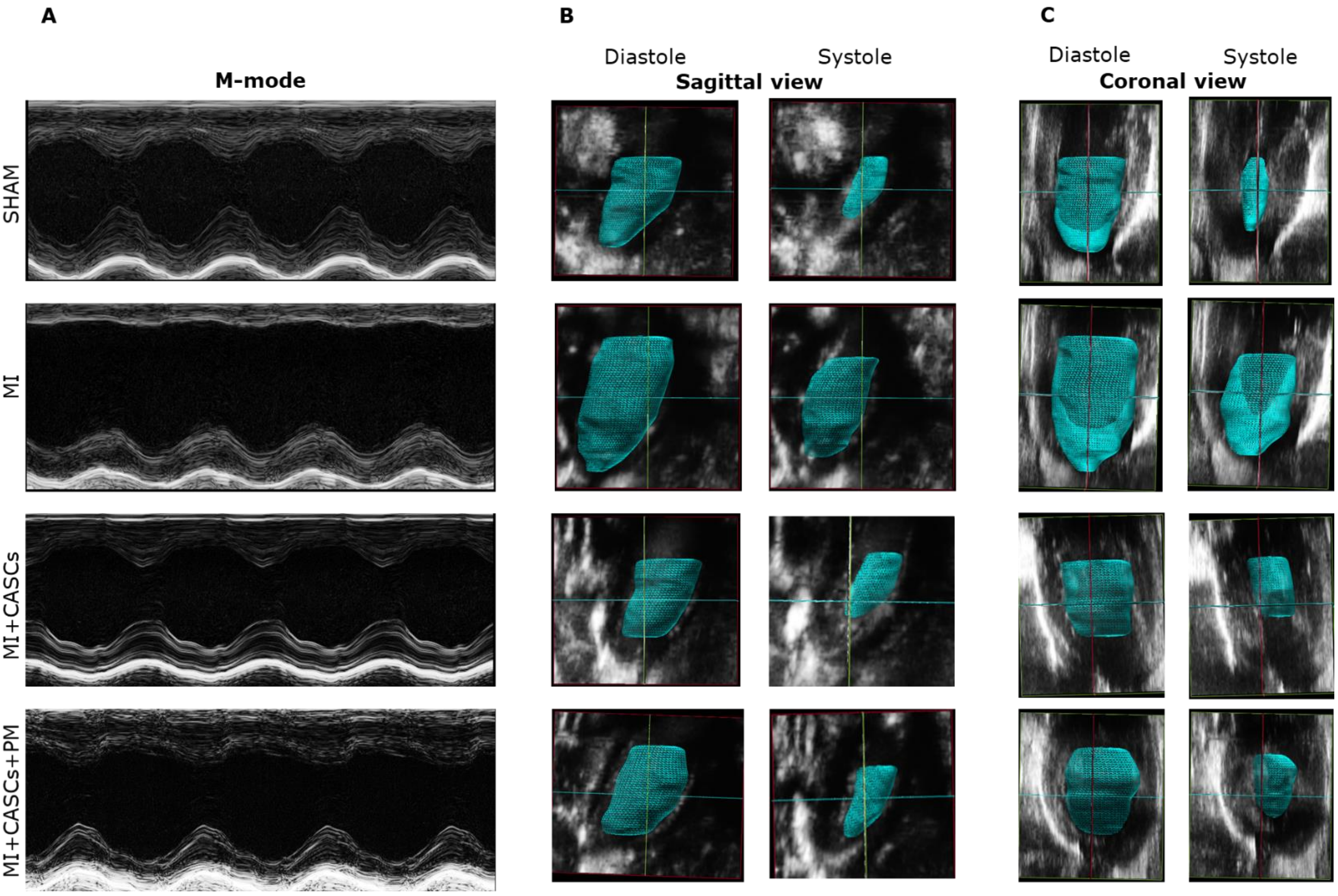

Figure S1. Representative echocardiographic images. (A) Representative images of M-modes obtained during parasternal short-axis view at mid-ventricular level of SHAM, MI, MI + CASCs, and MI + CASCs + PM animals. (B,C) Representative images of 4D reconstructed hearts during diastole and systole in (B) sagittal view and (C) coronal view of SHAM, MI, MI + CASCs, and MI + CASCs + PM animals.

Table S1: Diastolic echocardiographic characteristics.

**Parameters**	**4 Weeks Post-Operative**			
	SHAM	MI	MI + CASCs	MI + CASCs + PM
Longitudinal FS (%)	18 ± 3	18 ± 3	22 ± 2	23 ± 3
Radial FS (%)	50 ± 5	38 ± 5	50 ± 5	47 ± 4
E/E′	-26 ± 4	-32 ± 6	-29 ± 3	-27 ± 4
E/A	1.39 ± 0.21	1.80 ± 0.35	1.33 ± 0.11	1.27 ± 0.07
IVCT (ms)	21.1 ± 1.2	23.8 ± 1.1	22.3 ± 1.3	22.2 ± 1.3
IVRT (ms)	24.1 ± 1.5	22.7 ± 1.2	21.2 ± 1.0	22.2 ± 1.0

Diastolic echocardiographic characteristics 4 weeks post-op in SHAM (n = 5), MI (n = 11), MI + CASCs (n = 10), and MI + CASCs + PM (n = 9) animals. Data are expressed as mean ± SEM. FS: fractional shortening, E: early diastolic transmitral flow velocity, E′: peak early-diastolic annular velocity, A: late diastolic transmitral flow velocity, IVCT: isovolumetric contraction time, IVRT: isovolumetric relaxation time.

Table S2: Sequences of forward and reverse primers used in qPCR.

**Gene**	**Forward Primer**	**Reverse Primer**
IFN-γ	GAA AGA CAA CCA GGC CAT CAG	TCA TGA ATG CAT CCT TTT TTG C
IL-6	CGA AAG TCA ACT CCA TCT GCC	GGC AAC TGG CTG GAA GTC TCT
HPRT	CTC ATG GAC TGA TTA TGG ACA GGA C	GCA GGT CAG CAA AGA ACT TAA AGC C
PGK1	ATG CAA AGA CTG GCC AAG CTA C	AGC CAC AGC CTC AGC ATA TTC

Primer sequences are given in their 5′-3′ orientation. IFN-γ: interferon-γ, IL-6: interleukin-6, HPRT: hypoxanthine-guanine phosphoribosyl transferase, PGK1: phosphoglycerate kinase 1.
